# Two-Wired Active Spring-Loaded Dry Electrodes for EEG Measurements

**DOI:** 10.3390/s19204572

**Published:** 2019-10-21

**Authors:** Seungchan Lee, Younghak Shin, Anil Kumar, Kiseon Kim, Heung-No Lee

**Affiliations:** 1 School of Electrical Engineering and Computer Science, Gwangju Institute of Science and Technology, Gwangju 61005, Korea; futuremax7@gmail.com (S.L.); anilkdee@gmail.com (A.K.); kskim@gist.ac.kr (K.K.); 2 LG CNS AI&BigData Research Center, Seoul 07795, Korea; shinyh0919@gmail.com

**Keywords:** EEG measurements, active electrodes, spring-loaded dry electrodes, two-wired electrodes, bootstrapping topology

## Abstract

Dry contact electrode-based EEG acquisition is one of the easiest ways to obtain neural information from the human brain, providing many advantages such as rapid installation, and enhanced wearability. However, high contact impedance due to insufficient electrical coupling at the electrode-scalp interface still remains a critical issue. In this paper, a two-wired active dry electrode system is proposed by combining finger-shaped spring-loaded probes and active buffer circuits. The shrinkable probes and bootstrap topology-based buffer circuitry provide reliable electrical coupling with an uneven and hairy scalp and effective input impedance conversion along with low input capacitance. Through analysis of the equivalent circuit model, the proposed electrode was carefully designed by employing off-the-shelf discrete components and a low-noise zero-drift amplifier. Several electrical evaluations such as noise spectral density measurements and input capacitance estimation were performed together with simple experiments for alpha rhythm detection. The experimental results showed that the proposed electrode is capable of clear detection for the alpha rhythm activation, with excellent electrical characteristics such as low-noise of 1.131 μV_RMS_ and 32.3% reduction of input capacitance.

## 1. Introduction

During the last few decades, dry contact electrode-based electroencephalogram (EEG) acquisition [1] is one of the easiest ways to obtain neural information from the human brain in real time. This type of electrode is rapidly replacing conventional wet electrodes, which have been used in a variety of applications such as patient monitoring of neurological disorders [2], brain–computer interfaces [3], and biofeedback [4]. Nowadays, dry electrodes are integrated into portable commercial devices with wearable technologies to provide personal services such as healthcare and home diagnostics to improve the quality of life. These electrodes are designed to eliminate the need for electrolytic gels, which makes the installation process simple with a short setup time and also prevents an increase in impedance due to drying of gels. However, the absence of conductive gels means that controlling the contact impedance at the electrode–scalp interface is more difficult than using the conventional wet electrodes. Therefore, the impedance characteristics and the physical contact capability of the electrode device have become crucial design considerations for practical electrolyte-free EEG measurements.

Considering these design challenges, many researchers have endeavored to design better dry electrodes with various innovative ideas. Generally, these electrodes can be classified into three different categories: microelectromechanical systems (MEMS)-based electrodes, capacitive electrodes, and finger-shaped electrodes based on the probe shape and electrical coupling topology at the electrode–scalp interface.

In the MEMS-based dry electrodes [5,6], an array of microneedles are employed to penetrate the 10–40 μm thickness outer skin layer of the scalp. Spiky microneedles, which have lengths of 100–210 μm [7], around 150 μm [8], and 300 μm [9], are typically fabricated on a silicon wafer using special etching processes. In addition to silicon-based materials, a brush-type carbon nanotube-based [10], chitosan/Au-TiO_2_ nanotube-based [11], and polydimethylsiloxane (PDMS) substrate-based MEMS electrodes [12] have been developed for various kinds of electrophysiological sensing. Although the tip of microneedles can pass directly through into the inner skin layer to create a direct DC-coupled interface with the scalp surface, their complicated and costly fabrication process and infection risks still remain as practical constraints. In addition, EEG measurements on a hairy scalp are still limited because of the fragile and microscopic needles, which do not penetrate the hair layer effectively.

Capacitive electrodes are generally designed by building AC-coupled non-contact interfaces between the scalp surface and electrodes, utilizing insulation materials such as a hair layer, cotton fabric or printed circuit board (PCB) [13]. This AC-coupled interface can function as a capacitor on the electrode frontend, due to which the acquired biopotentials pass across the electrically insulated layer. With regard to this, Sullivan et al. [14] and Chi et al. [15,16] have proposed PCB plate-based capacitive electrodes equipped with discrete off-the-shelf components or a customized application-specific integrated circuit (ASIC). Capacitive electrodes based on soft insulating materials such as polymer foam [17], PDMS [18] and carbon nanotube [19] have also been introduced. However, there are still many design issues related to measurement distortion such as gain attenuation and phase drift due to the AC-coupled interface [20].

Finger-shaped dry electrodes have also been developed for direct-contact biopotential measurements. In these electrodes, the shape of the probe part has been designed to penetrate the hair layer; therefore, DC-coupled interfaces can be easily made by touching the probes to the scalp surface. From this idea, a shrinkable spring-loaded probe-based passive dry electrode [21], a brush-type flexible dry electrode [22,23], a pin-shaped conductive polymer-based dry electrode [24,25] and a 3D-printed dry-fingered electrode [26] have also been proposed. However, high and unstable contact impedance due to the electrolyte-free interface remains a major challenge in this type of dry electrode.

One possible approach to solve this issue is that the electrode device itself supplies conductive liquid to lower the contact impedance. This method has been presented in the literature [27], but the semi-dry approach still has some of the same problems as the wet types. Another approach is to embed supplementary active circuitry in the electrode device to electronically maximize the input impedance characteristics of the dry electrodes. Following this approach, active electrodes [28] with various circuit topologies designed using off-the-shelf discrete components [29,30] and ASICs [15,31,32] have been proposed. This overall research trend suggests that dry electrodes require that the electrode device is able to make reliable contact with the scalp surface and have high input impedance characteristics.

To meet these requirements, this study proposes a 2-wired active spring-loaded dry electrode to simultaneously achieve high-precision and electrolyte-free EEG monitoring. The proposed electrode is designed with a combination of spring-loaded probes and an active buffer circuit. The finger-shaped probes are able to penetrate the hairs on the scalp without prior preparation, and their shrinkable spring-loaded structures provide mechanical flexibility to each probe for adjustable contact intensity along the curvature of the uneven scalp surface. These structural advantages effectively improve the contact efficiencies of the electrodes with the scalp surface. The zero-drift amplifier-based active buffer circuit provides low-noise impedance conversion to stabilize the intractable impedance characteristics of the dry electrodes caused by the absence of the conductive paste. In the design of the active circuit, the 2-wired bootstrap topology reduces the number of wire connections and provides further enhancement of the input impedance by reducing the input capacitance.

To achieve low-noise and attenuation-free EEG measurements, an equivalent circuit model and amplifier requirements for active circuits were theoretically analyzed in the design process. Evaluations of the electrical characteristics such as spectral noise power density and input capacitance were also performed along with a simple alpha rhythm detection test to verify the EEG feature detection capability.

The contribution of this study is to present an optimized design for an active dry electrode for EEG measurements by combining the electronically maximized impedance characteristic and the physically maximized contact capability of the electrode device.

The remainder of the paper is organized as follows: Section 2 provides detailed descriptions of the design and implementation methods along with an electrical analysis of the equivalent circuit model. The evaluation of the electrical characteristics as well as the experimental methodology for alpha rhythm detection is presented in Section 3. Section 4 summarizes several results, including the evaluation of the electrical characteristics and alpha rhythm detection capability. Finally, a brief discussion of this study and a summary of the proposed electrode development are given in Section 5 and Section 6.

## 2. Design and Implementation

### 2.1. Two-Wired Active Electrode Design

Active electrodes require an active power supply. At least three wired connections are needed, instead of a single wire, for both the power supply and signal transmission. Compared to conventional passive electrodes that do not require a power supply, the additional wires make it difficult to handle rigid wires and increase the design complexity of the biopotential acquisition system. To reduce the number of wires for the active electrodes, a bootstrap technique [33] was employed for the proposed active dry electrodes. This technique reduces the number of electrode wires by replacing the conventional voltage-based power supply with a current source-based power supply. The power supply rails and signal transmission lines can be shared over a single wire, resulting in an active electrode design that requires only two wire connections.

**Figure 1:**
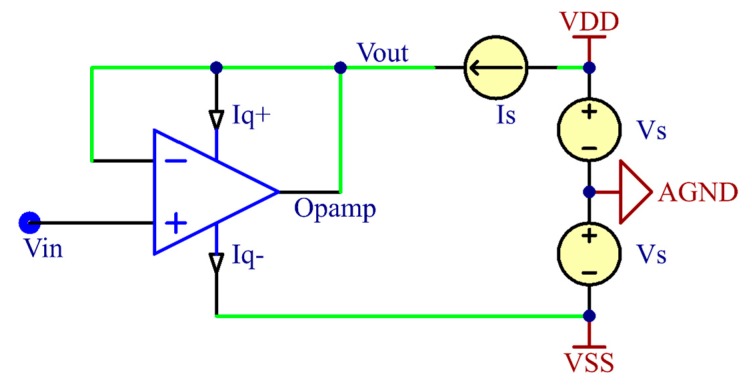
Simplified schematic of bipolar two-wired active electrode with bootstrapping topology.

Figure 1 shows the simplified schematic of the bootstrap technique-based active electrode system using an operational amplifier buffer. The half-power supply bootstrap scheme is implemented by connecting the amplifier’s positive power supply rail with its signal output node to a current source. At this point, the current source $I_{s}$ feeds current to the positive power rail of the amplifier, while the signal output node of the amplifier consumes the surplus current. The signal output voltage is therefore determined as follows:

(1) $$V_{o}=\frac{(I_{s}-I_{q+})R_{o}+A_{ol}V_{i}}{A_{ol}+1}≃V_{i}$$

where $A_{ol}$, $R_{o}$, and $I_{q+}$ are the amplifier’s open-loop gain, output impedance, and quiescent current on the positive power supply rail, respectively.

Generally, the open-loop gain of an amplifier is very large, so the current biasing effects on the output node are neglected. Therefore, the output node voltage will be followed to the input node voltage, and the bootstrapped wire connected with output node can then be used as a signal output link for the active electrode system. However, this circuit design lowers the voltage delivered to the amplifier’s positive power supply rail unintentionally, making it difficult to meet the minimum operating voltage for normal amplifier operation in some special cases. To avoid this cases, the operating voltage range of the amplifier needs to be checked. This requirement is discussed further in Section 2.3.2.

The unity-gain buffer configuration allows transformation from the low impedance of the biopotential source to the possible highest impedance [34]. Because the input impedance of the buffer circuit is determined as the differential input impedance multiplied by the open-loop gain, this configuration enables maximizing the electrode impedance. The extremely high input impedance of the dry electrode enables virtually perfected isolation between the source and load, and thus eliminates the loading effects. This property helps to provide a robust signal, which is hardly affected by motion artifacts and power line interferences.

### 2.2. Electrical Model Analysis and Design Considerations

**Figure 2:**
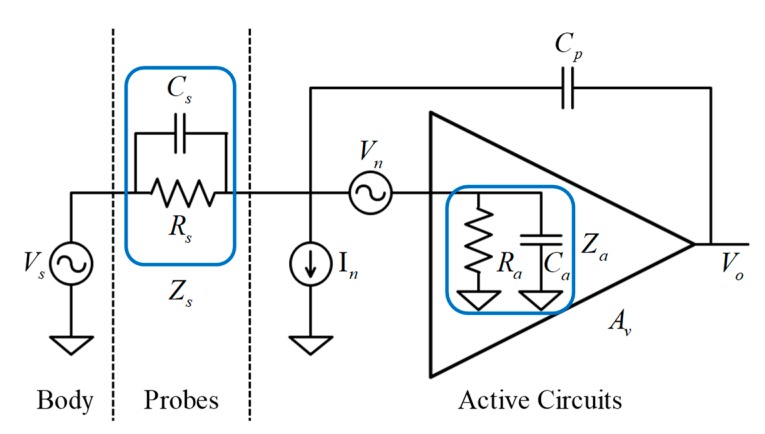
Equivalent circuit model of the proposed active spring-loaded electrodes.

To investigate the electrical characteristics such as source-to-output gain and input-referred noise of the active circuits, we analyzed the electrical coupling model of the skin–electrode interface for the proposed active circuit. A general electrical model of the active electrode circuit was analytically studied by Chi [13]. Figure 2 shows an equivalent electrical model of the proposed active dry electrode reinterpreted from the general active electrode model. In this circuit model, $V_{s}$ and $V_{o}$ denote the biopotential source generated from the human brain and output node of the active circuit, respectively. $R_{s}$ and $C_{s}$ represent the resistive and capacitive properties of the scalp-electrode interface established by dry contact of the spring-loaded probes, respectively. $R_{a}$ and $C_{a}$ indicate the input resistance and capacitance of the amplifier, respectively. $C_{p}$ denotes the parasitic capacitance [35] originating from the voltage difference between the signal input and output through active shielding. $A_{v}$ is the gain of the circuit and is set to unity because the proposed active circuit is designed to operate under a buffer configuration. In order to easily calculate the gain and input-referred noise of the circuit model, the resistances and capacitances have been substituted in parallel at the interface layer ($R_{s}//C_{s}$) and input node of the amplifier ($R_{a}//C_{a}$) for impedance $Z_{s}$ and $Z_{a}$, respectively. Using nodal analysis, the formulation for source-to-output gain of the equivalent circuit model can be derived as:

(2) $$\begin{array}{cc} G\left(jw\right)=\frac{V_{o}}{V_{s}} & =\frac{Z_{a}}{Z_{s}+Z_{a}+(1-A_{v})jwC_{p}Z_{s}Z_{a}}\\ & =\frac{R_{a}(jwR_{s}C_{s}+1)}{jwR_{s}R_{a}[C_{a}+C_{s}+(1-A_{v})C_{p}]+R_{s}+R_{a}} \end{array}$$

With a low-frequency biopotential source, the contributions of the resistive components are relatively high because of the reduction of the w factor. In the extreme DC case, where w is equal to zero, this gain formula simply changes to $R_{a}/(R_{s}+R_{a})$. As the value of $R_{a}$ increases, $R_{s}$ becomes negligible, which means that the input impedance specification of the amplifier directly affects the gain attenuation of the low-frequency biopotential source.

Conversely, with a high-frequency biopotential source, the contribution of the capacitive components increases. Hence, $C_{s}$ needs to be maximized, while $C_{a}$ and $C_{p}$ need to be minimized in order to avoid gain attenuation of the high-frequency biopotential source. $C_{p}$ can be minimized by suppressing the leakage current between the input and output nodes. This can be achieved by shielding the input node with the output node of the same potential as the input node. $C_{a}$ is the amplifier’s internal parasitic capacitance that originates from between the input node and both of the power supply rails [36]. Thus, this parasitic capacitance can be considered as a combination of the capacitance built up between the input node and the positive rail ($C_{a+}$) and between the input node and negative rail ($C_{a-}$). Applying the bootstrap topology to the proposed active circuit, the voltage difference between the signal output node, which has the same potential as the signal input node, and the positive voltage supply rail of the amplifier can be minimized. Therefore, $C_{a+}$ can be effectively eliminated, and the total capacitance of $C_{a}$ can also be minimized. $C_{s}$ is involved in the electrode contact efficiency with the scalp surface. When using non-flexible rigid probes, it is difficult to achieve tight contact with the scalp, resulting in an air gap between the probes and scalp surface. This air gap is equivalent to another extra capacitor, which is connected with $C_{s}$ in series. Consequently, the total capacitance of $C_{s}$ will be reduced because of the series connection of two individual capacitors. The flexible spring-loaded probes, on the other hand, can easily adjust their contact intensities in accordance with the curvature of the scalp surface, thus preventing to the building of air gaps. Therefore, the maximization of $C_{s}$ can be achieved by employing spring-loaded probes.

To quantitatively analyze the noise performance of the active circuit, the noise voltage with respect to the biopotential source input can be expressed as:

(3) $$N_{in}=\left(\frac{Z_{s}+Z_{a}+jwC_{p}Z_{s}Z_{a}}{Z_{a}}\right)V_{n}+Z_{s}I_{n}$$

and the power density, which is equal to the root-mean-squared (RMS) power of the input-referred noise voltage, can also be derived as:

(4) $$N_{in,rms}^{2}=\left(\frac{{\left|Z_{s}+Z_{a}+jwC_{p}Z_{s}Z_{a}\right|}^{2}}{{\left|Z_{a}\right|}^{2}}\right)V_{n,rms}^{2}+{\left|Z_{s}\right|}^{2}I_{n,rms}^{2}$$

where $V_{n,rms}^{2}$ and $I_{n,rms}^{2}$ denote the RMS-squared power of the voltage and current noise sources $V_{n}$ and $I_{n}$, respectively. These noise sources are derived from the noise model of the amplifier [37], and these parameters depend on the electrical characteristics specified in the amplifier datasheet. Therefore, amplifier selection is a key optimization factor for low-noise biopotential acquisition, and it will be discussed in Section 2.3.2.

For low input-referred noise performance, it is obvious that the operand terms multiplied with the voltage and current noise sources need to be minimized. To lower the voltage noise $V_{n}$, $Z_{a}$ firstly needs to be maximized. The bootstrapping topology provides low input capacitance characteristics by reducing the parasitic capacitance of the amplifier, resulting in high input impedance of the amplifiers. $C_{p}$ should also be minimized for further reduction of the voltage noise term, which can be achieved by preventing leakage current with robust shielding of the input node. The current noise is typically dominated by the scalp–electrode coupling impedance $Z_{s}$, which is inversely proportional to the electrode contact efficiency. To lower the current noise $I_{n}$, high contact efficiency is required, meaning that low coupling impedance with low resistance and high capacitance must be achieved. These requirements can be achieved by equipping multiple spring-loaded probes in the design of the proposed electrode. Installation of the twelve parallelly connected probes lowers the resistive impedance, which in turn prevents poor electrical coupling caused by loose installation of the electrode unit. In addition, the probe’s shrinkable structure fills the air gaps caused by microcontact failures at the scalp–electrode interface, thereby continuously keeping high capacitance characteristics.

### 2.3. Design of Active Dry Electrodes

#### 2.3.1. Spring-Loaded Probes

The EEG signals are acquired using the spring-loaded probes (SK100 Series [19], Leeno Industrial Inc., Pusan, Korea) by contact with the scalp surface. In each electrode, a total of 12 probes are soldered to the active circuit PCB, inside the electrode. Each probe consists of four components: plunger, barrel, spring, and probe receptacles. The plunger has a cylindrical shape, which is coated with beryllium copper and gold plated over nickel. The plating materials are biocompatible, and they do not induce any allergic reactions. The tip of the plunger, which is in contact with the scalp, has a round shape to minimize stabbing pain. The plunger is combined with a barrel and spring to make a spring-loaded structure. With the aid of the embedded springs, the probe is shrinkable up to a maximum of 1.5 mm along with the barrel. The initial pressure of the spring is only 10 g in the preloaded state of the probe. When the probe shrinks to its minimum length, the spring delivers up to 54 g of pressure to the scalp. Therefore, this linearly increasing force ensures that appropriate contact pressure continues to be applied along the uneven scalp surface. The probes also relieve pain by absorbing the excess pressure in the vertical direction. In the electrical specifications, the resistance of each probe is given as less than 50 mΩ, which is sufficiently low for conducting biopotential signals.

#### 2.3.2. Amplifier Specifications

With reference to the electrical model analyses for the proposed active circuits in Section 2.2, it can be observed that the input-referred noise is primarily affected by the electrical specifications of the amplifier. In addition to this, the circuit design with bootstrap topology lowers the voltage on the positive power supply rail in proportion to the input biopotential voltage, which may not meet the minimum voltage requirement for normal amplifier operation. The other amplifier specifications, including offset voltage, input bias current, and quiescent current, should also be checked for the DC-coupled circuit design and longer operation times.

To fulfill these particular requirements, an OPA378 operational amplifier (Texas Instruments, Dallas, TX, USA) [38] was employed for the proposed electrodes. This amplifier provides outstanding characteristics such as low-noise, minimal input offset, a wide acceptable range of power supply voltages, and low power consumption optimized for battery-powered medical instruments. These key parameters are summarized in Table 1.

Because of the microscopic amplitudes of the EEGs, the noise characteristic is the most important parameter in the design of a biopotential sensor, which is indicated as the noise voltage and its spectral densities in the datasheet. According to the IEC standard [28], input-referred noise below 6 μV_PP_ is acceptable for EEG acquisition systems, and the OPA378 fulfills this condition.

For low-noise EEG measurements in the frequency bands near DC, the offset voltage and its drift need to be checked because they represent measurement precision at the DC region. In low-frequency bands close to DC, 1/f noise, called flicker noise [39], is more dominate than other type noises. This type of noise is amplified when approaching the DC region, for which the noise power spectral density is inversely proportional to the square root of the frequency, making it a major noise contributor to the low-frequency band near DC. When a large DC offset is coupled directly with the input of the EEG acquisition system, it can saturate the high-gain preamplifiers and diminish their dynamic range. To mitigate the DC offset, operational amplifiers equipped with advanced circuit design techniques such as auto-calibration and chopping have been introduced and are known as zero-drift amplifiers [28,40]. Utilizing the auto-calibration technique, a signal pathway of the OPA378 continuously corrects the incoming offset voltage every 3 μs with a 350 kHz sample-and-hold circuit. Therefore, this auto-calibration technique maintains a noise voltage density of 20 nV down to 1 Hz and achieves a noise voltage of 0.4 μV_PP_ in the bandwidth of 0.1–10 Hz, thereby extending the acceptable low-frequency range of measurements without an AC-coupled high-pass filter.

As mentioned in Section 2.1, the bootstrap topology lowers the input capacitance of the amplifier by connecting its positive power supply rail to the signal output node, while also lowering the range of voltages supplied to the amplifier. Normal operation cannot be guaranteed, when the supply voltage range does not meet the minimum voltage requirement of 2.2 V. Based on the proposed circuit design, as the voltage of −2.5 V is already supplied to the negative power rail of the amplifier, the common-mode voltage of the input biopotential should be kept at least −0.3 V to meet the minimum voltage requirement. This condition is practically unlikely because of the stable offset characteristics of the amplifier. Nonetheless, if the common-mode voltage of the input node drops below −0.3, the amplifier will be turned off, and thereby the output node of the circuit can be left in a floating state.

Moreover, an on-chip electromagnetic interference (EMI) filter with 25 MHz cutoff frequency provides outstanding EMI suppression. This feature prevents offset shifts in the amplifier output caused by EMI and allows more precise measurements. The low current consumption of up to 150 μA easily enables multichannel and battery-powered instrumentation.

#### 2.3.3. Circuit Design and Implementation

**Figure 3:**
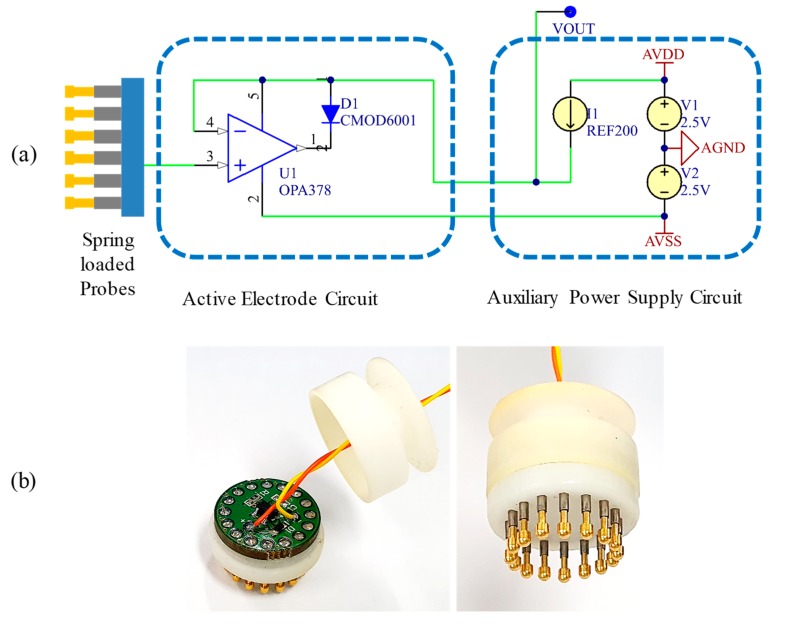
Actual implemented schematic (**a**) and images (**b**) of the proposed active dry electrode. The proposed electrode system comprises the electrode unit itself and an auxiliary circuit board for the voltage and current power supplies. In the electrical schematic, decoupling capacitors for stabilized voltage supplies are omitted for simplicity.

The schematic of the proposed active dry electrode and its prototype images are shown in Figure 3a,b, respectively. The proposed system comprises two individual parts—the electrode unit and auxiliary board.

The electrode unit is cylindrical in shape with a diameter of 11 mm and a height of 17 mm. The electrode is composed of the 12 spring-loaded probes, OPA378 amplifier, and CMOD6001 low-leakage diode (Central Semiconductor, Hauppauge, NY, USA), and these are installed in the electrode PCB embedded in the 3D-printed electrode housing. All probes are electrically connected to each other, and the measured biopotentials are delivered to the input node of the amplifier. The buffered biopotentials are then finally transferred to the auxiliary board through the bootstrapped wire, which is connected to the current-sourcing device. Concurrently, this current-sourcing device in the auxiliary board supplies a bias current for the amplifier operation through the same bootstrapped wire. The diode is inserted between the amplifier output and positive rail to keep the output voltage swing lower than that of the positive rail by the inherent forward voltage drop of the diode [41]. Even though the amplifier supports rail-to-rail output that allows maximizing the output swing over the entire range of the supply voltage, this diode is necessary to keep an extra margin for low-distortion voltage output and low power consumption.

The auxiliary board is designed to provide a constant current source and bipolar voltage power using linear regulators, current source devices, and numerous decoupling capacitors. To supply low-noise voltage for the +2.5 V and −2.5 V rails, ADM7154 and ADP7183 linear regulators (Analog Devices, Norwood, MA, USA) were used. These regulators provide extremely low-noise performance of 1.6 μV_RMS_ and 4 μV_RMS_ along with high power supply rejection ratios, which are optimized for noise-sensitive applications. A REF200 (Texas Instruments, Dallas, TX, USA) [42], which is embedded with two 100 μA current sources, was used as the current-sourcing device. By connecting the regulated 2.5 V rail to the current sources, the device is capable of simultaneously powering two channels of the proposed electrodes. Although the current-sourcing capability is limited to 100 μA per channel, the current requirement for the positive rail of the amplifier is only 75 μA, which is half of the maximum current consumption of 150 μA, thus ensuring sufficient current supply. All electrical components are small and surface mounted type, thereby making it easy to design portable size instruments.

## 3. Evaluation and Experiment

### 3.1. Noise Characteristics

In the design of electronic circuit-based sensors, the noise floor of the sensing signals is a key parameter for determining the integrity of the acquired data. To evaluate the noise characteristics of the proposed active electrode circuit, noise power spectral densities were analyzed using an FFT-based spectrum analyzer (Keysight 35670A, Santa Rosa, CA, USA), which can quickly capture the spectral information of analog signals utilizing Fourier analysis and digital signal processing techniques. With this instrument, the total noise output of a circuit can be estimated by shorting the circuit’s input node to ground potential and measuring the power spectral densities at the output node of the circuit. To compare the noise measurements of the proposed 2-wired bootstrap buffered circuit, a bipolar-powered 3-wired conventional buffered circuit was implemented as a target of comparison. For the two types of active circuits, 1600-point power spectral densities were measured over the 0.1–200 Hz bandwidth. These measurements were repeated 50 times and averaged for a smoother representation. The measured noise spectra were transmitted to a laptop using a USB-type GPIB interface and instrument control software (Keysight VEE Pro 9.2, Santa Rosa, CA, USA). To reject noise interference, this evaluation was performed within an aluminum enclosure.

In the analysis stage, Pearson correlation coefficients and Wilcoxon signed-rank test was used to measure orientational and statistical similarities between the two pairs of noise spectral densities. To compare actual noise voltages in the EEG bandwidth precisely, RMS voltages were also calculated from the measured noise spectral densities by taking the squared values of the given voltage spectral density, integrating within the specified frequency range, and computing the square root.

### 3.2. Input Capacitance

In the electrode design for EEG measurements, high input impedance is an essential characteristic for further signal conditioning processes. High input impedance also implies low input capacitance at higher frequencies. To investigate the impedance characteristics of the proposed electrode circuit, the input capacitances for the proposed circuit (2-wired bootstrap buffered circuit) and its counterpart (3-wired conventional buffered circuit) were analyzed. Since the input capacitance of the operational amplifier is typically lower than a few picofarads, the direct measurements for observing input capacitance using a multimeter are not practical because of its poor error tolerance. In order to measure the input capacitance of the operational amplifier-based circuit, a large resistor was inserted in series with the input node of the amplifier. This configuration set up a first-order RC lowpass filter in combination with the internal capacitance of the amplifier. Through the frequency response analysis for the circuits, the input capacitances can be inversely estimated by evaluating the −3 dB cutoff frequencies. Detailed information on this methodology is described in [43].

The same spectrum analyzer was used to investigate the input-to-output frequency responses for the test circuits. After inserting a 2 MΩ resistor as a large source resistor $R_{s}$, a 100 mV_pp_ sinusoidal sweep was applied to the input node of the target circuit in accordance with 800 log-scaled bins arranged over the 1–51.2 kHz bandwidth. The swept source was routed to the input probe of the spectrum analyzer using a signal splitter, while the output probe was connected to the output node of the target circuit, unlike the test setup in [43]. This is because a unity-gain buffer configuration allows the input signal of the amplifier to be identically measured at the output node of the circuit without the need for a high-impedance FET probe. From this setup, dB-scaled Bode plots can be obtained from the frequency analyses, and we can estimate the input capacitance of the test circuit using the following equation: $C_{in}=1/(2\pi R_{s}f_{-3dB})$. All tests were carried out using customized test PCBs that were carefully designed with active shielding to avoid other parasitic capacitances.

### 3.3. Alpha Rhythm Detection Experiment

Alpha rhythm, the most prominent feature of an EEG, can be easily utilized as a benchmark tool for testing the detection capabilities of real EEG features. When users close their eyes, the spectral power of the alpha rhythm band (8–15 Hz) is amplified compared to other spectral ranges, and vice versa when the users open their eyes. By comparing the spectral activation for the alpha rhythm when the eyes are closed or open, we can evaluate the practical applicability of the proposed electrode for real EEG monitoring.

For this purpose, ten trials were performed for a subject. A single trial consisted of maintaining the eye-open state for 12.5 ± 2.5 s and the eye-closed state for 10 s. For every transition of instruction, a beep sound was used to inform the subject of the command changes. Alternative electrodes such as a 3-wired active buffered electrode and a passive dry electrode, as well as the proposed electrode, were employed for the comparison of EEG measurements. All electrode implementations were equipped with the same spring-loaded probe for dry contact with the scalp surface. These three electrodes were installed as close as possible to the Fz position according to the international 10–20 system. Disposable wet electrodes were also attached to the skin behind the left and right earlobes as a reference and a bias electrode, respectively. Experiments were conducted using MATLAB 2014a (Mathworks, Natick, MA, USA) and the Cogent 2000 toolbox, and EEG measurements were recorded using the ADS1299-based EEG acquisition system which was built in a previous study [44].

Offline analyses for the EEG measurements were also performed using MATLAB 2014a. The raw EEG dataset was filtered with a 4th order zero-phase 0.5–40 Hz bandpass Butterworth filter. From the filtered EEG dataset, the epochs for 5 s corresponding to each condition were extracted based on the recorded event triggers. The spectral power values were also calculated to precisely compare the spectral activation for the alpha rhythm. The 10-s EEG measurements were also visualized before and after the fifth eye-close instruction for time-series waveform comparisons. In addition, the similarity of the bandpass-filtered waveforms was evaluated in terms of Pearson correlation coefficients.

## 4. Results

### 4.1. Noise Power Spectral Density

**Figure 4:**
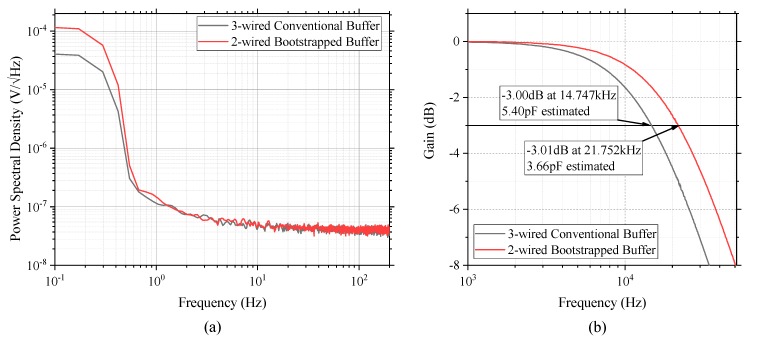
(**a**) Measurements of the noise power spectral densities and (**b**) input capacitance estimation results for the proposed active electrode circuit and its alternative implementation (2-wired bootstrapped buffered topology vs. 3-wired conventional buffered topology).

The comparison of the noise power spectral densities for the proposed active electrode circuit and its conventional counterpart are depicted in Figure 4a. Compared to the noise power spectral densities of the conventional 3-wired buffer circuit, the proposed 2-wired bootstrap topology shows a similar trend along with a correlation coefficient of 0.953 within the EEG bandwidth of 0.5–50 Hz. However, in the Wilcoxon signed-rank test, a nonparametric statistical method for testing a hypothesis of paired data, the two paired noise spectral densities do not show statistical similarities with a low significant level (p < 0.0001) at the same EEG bandwidth. In a complementary analysis for a sum of the difference between the paired noise spectral densities, we found that the proposed design produces more noise by 2.0433 nV√Hz on average than the 3-wired counterpart. Consequently, this extra noise leads to a small difference between the estimated RMS noise voltages (i.e., 1.131 μV_RMS_ with the proposed 2-wired topology vs. 1.017 μV_RMS_ with the 3-wired counterpart). The slightly increased RMS noise in the proposed topology is due to an increase in noise power at lower frequency bands below 1 Hz. The reason for this is the positive rail voltage of the proposed topology, which has continuously changed in accordance with the voltage corresponding to the acquired input signal, instead of being supplied from a low-noise constant voltage source. This unfixed supply voltage, combined with thermal noise and other interference, seems to result in minor extra noise in the low-frequency region.

### 4.2. Input Capacitance Estimation

The spectral analysis results for investigation of the −3 dB cutoff frequencies and the estimated input capacitance from the results are depicted in Figure 4b. For the two types of circuit configuration, the differences in the cutoff frequencies is about 7 kHz, resulting in a 1.74 pF reduction in the input capacitance for the proposed bootstrap configuration compared to the conventional buffer design approach. The impedance of the amplifier is represented as R/(jwRC+1), because it is simplified as a parallel combination of resistance and capacitance. Therefore, an approximately 32.2% reduction in the input capacitance leads to roughly 147.5% impedance boosting within the EEG bandwidth. This impedance boosting effect makes the measurement more robust against artifacts and EMI interference.

### 4.3. Experimental Results of Alpha Rhythm Detection

**Figure 5:**
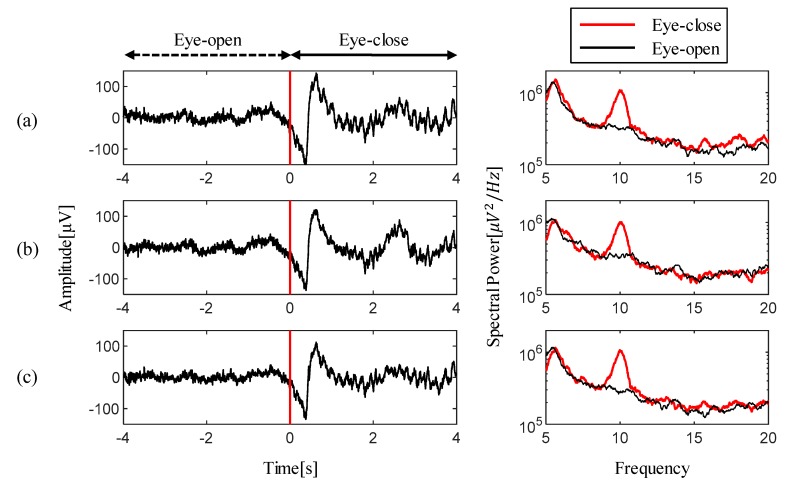
EEG measurements and their spectral comparisons for (**a**) proposed 2-wired active dry electrode, (**b**) alternative 3-wired active dry electrode, and (**c**) passive dry electrode. On the left, the red vertical line on the EEGs indicate the task onset timing for the eye-close instructions. During the eye-close session, activated alpha waves are commonly observed in the time-series and spectral visualization results for all types of electrodes.

Figure 5 shows the experimental results for the alpha rhythm detection test measured by three types (2-wired active, 3-wired active, and passive) of dry electrodes. The captured time-series waveforms on the left side of figures were extracted from the EEG measurements near the onset time of the fifth task from among the 10 trials. In these waveforms, a red vertical line indicates the start time for the eye-close instruction. Within one second after task onset, slightly large voltage swings were observed in all measurements while the subject’s eyelids are closed. After the swings, it was confirmed that clear alpha waves were identified with their distinguished oscillations of measured waveforms. These evoked alpha waves are easily noticeable in the spectral analysis. The figures on the right side show the results of the event-related spectral analysis for each electrode measurements. These spectral comparisons clearly visualize the maximized spectral differences evoked near the 10 Hz, which belong to the alpha rhythm. Specifically, the maximum spectral differences for two different tasks were observed at 12.4 dB at 10.1 Hz for the proposed 2-wired active electrode, 11.28 dB at 10.06 Hz for the 3-wired active electrode, and 13.83 dB at 9.98 Hz for the passive electrode. These spectral analysis results confirmed that EEG feature detection can be fully achieved using the proposed electrode.

The comparison of the correlation coefficients for each paired EEG waveforms is summarized in Table 2. The correlation coefficient between the EEG waveforms using the passive dry electrode and the proposed 2-wired topology is ρ_2_; the correlation coefficient between the EEG waveforms using the passive dry electrode and the 3-wired counterpart is ρ_3_; and the correlation coefficient between the proposed 2-wired topology and the 3-wired counterpart is ρ_23_. An insignificant difference between ρ_2_ and ρ_3_ indicates that the proposed 2-wired electrode is sufficient to achieve measurements nearly equivalent to the conventional 3-wired design approach. A slight decrease in the value ρ_23_, compared to ρ_2_ and ρ_3_, is supposed to be caused by the difference in common-mode voltages in accordance with a difference in the design topology.

## 5. Discussion

Theoretical analysis of the equivalent circuit model for the proposed electrode indicated that the electrical specifications of the amplifier have a significant effect on measurement characteristics such as input-referred noise and gain attenuation. As standard specifications in the datasheet, the offset voltage and the 0.1–10 Hz peak-to-peak noise voltage are involved with not only the precision of the common-mode voltages, but also noise characteristics within the low-frequency bands near DC, associated with 1/f noise. Since even EEG waves with very low-frequency bands (0.1–4 Hz), including delta waves and slow oscillations, are often used for sleep studies [45], the examination of these specifications is required to verify the 1/f noise characteristics. The OPA378, a zero-drift amplifier with 0.1–10 Hz RMS noise of 0.4 μV and offset voltage of 20 μV, provides excellent low-noise characteristics, but noise boosting is still observed at lower frequencies below 1Hz in the actual noise measurements. This is because the 1/f noise is generated internally from the quantum mechanical random process inherent in all semiconductor devices, including the amplifier to be measured and the measurement instrument itself. This means it is difficult to eliminate 1/f noise completely. Nevertheless, the proposed electrode still presented excellent low noise characteristics of 1.131 μV_RMS_ within an EEG bandwidth of 0.5–50 Hz along with noise power spectral densities of 139 nV/√Hz at 1 Hz and 49 nV/√Hz at 10 Hz. These measurements are comparable with previous studies (7.4 μV_RMS_ within a bandwidth of 1–1000 Hz in the [41], and 200 nV/√Hz at 1Hz in the [15]).

On the other side, the ratio of the noise characteristics versus power consumption also needs to be checked to consider the entire power consumption of the EEG acquisition system. There is a trade-off relationship between power consumption and noise performance [46], which means that increased power consumption of the amplifier results in better low-noise characteristics in general. Amplifiers that require higher power can be used in the active electrodes for better noise performance, but this results in an increase in the overall power requirement of the instrument with numerous channels. For example, the state-of-the-art operational amplifier OPA188 (Texas Instruments, Dallas, TX, USA) exhibits a better noise voltage of 250 nV_PP_ over the 0.1–10 Hz bandwidth, which is a 37.5% lower noise voltage compared to that of the OPA378. However, its current consumption is typically increased 3.6 times more to 450 μA. The proposed electrode is designed to consume up to 150 μA of current per channel, resulting in a total of only 2.4 mA for 16 channels, thus it can be continuously operated for about 40 hours even with a 100 mAh small lithium polymer battery. This low-power operation adequately meets a design requirement for battery-powered mobile instruments.

Compared with previous studies [18,20,29], another difference in the frontend circuit design is the exclusion of a bias current path. In those previous studies, a large value resistor in the TΩ range or parallel connection of two reverse diodes is generally used as the bias current path. This is necessary to prevent voltage saturation at the input node of the amplifier caused by incoming bias currents, but it also generates a lot of thermal noise due to the high physical resistance. The problem here is that irrespective of the resistance value, degradation of the amplifier’s input impedance cannot be avoided. The proposed electrode omits the design of the bias current path, but the built-in protection circuitries embedded in the amplifier can fulfill this role to effectively prevent electrical overstress at the input node and degradation of the high input impedance.

## 6. Conclusions

In this study, we have proposed a two-wired active spring-loaded dry electrode to conduct electrolyte-free EEG monitoring. By combining spring-loaded probes with the active buffer circuit, the proposed electrode design simultaneously enables electronically maximized input impedance, and physically maximized contact capability. In the design process, the equivalent circuit model for the electrode circuit and its associated electrical parameters such as noise and gain attenuation were analyzed to obtain low-noise and attenuation-free EEG measurements. Based on the analysis, the active circuit was designed based on low-cost discrete components and the low-noise and low-offset zero-drift amplifier. The complete electrode device was implemented by combining the active buffer circuit with spring-loaded probes and a 3D-printed housing. Through several evaluations included the alpha rhythm detection test, the proposed electrodes were found to have a low-noise characteristic of 1.131 μV_RMS_ within the EEG bandwidth of 0.5–50Hz and the capability to clearly detect an alpha rhythm near 10 Hz. In addition, by applying the bootstrap topology to the proposed electrode design, the proposed electrode only requires a two-wired connection with an approximate 32.2% reduction in the input capacitance. This leads to an impedance boosting of roughly 147.5% within the EEG bandwidth. In our future work, we plan to design a portable instrument for mobile EEG monitoring based on the proposed electrode system.

## Figures and Tables

**Table 1:** Electrical characteristics of the OPA378 operational amplifier.

Electrical Parameters	Characteristics
Voltage noise	0.4 μV_PP_ at 0.1–10 Hz
Noise power spectral density	20 nV/√Hz at 1 kHz
Offset voltage and offset drift	20 μV and 0.1 μV/℃
Input capacitance	5 pF with common mode
Input bias current	± 150 pA, max. 550 pA
Power supply voltage range	2.2–5.5 V (rail-to-rail)
Quiescent current	125 μA, max. 150 μV

**Table 2:** Comparison of correlation coefficients for each paired EEG datasets.

2-wired Active vs. Passive (ρ_2_)	3-wired Active vs. Passive (ρ_3_)	2-wired Active vs. 3-wired Active (ρ_23_)
0.8536	0.8657	0.7854
